# [Retracted] Ginsenoside Rg3 ameliorates acute pancreatitis by activating the NRF2/HO-1-mediated ferroptosis pathway

**DOI:** 10.3892/ijmm.2024.5449

**Published:** 2024-10-29

**Authors:** Yuqiang Shan, Jiaotao Li, Akao Zhu, Wencheng Kong, Rongchao Ying, Weiming Zhu

Int J Mol Med 50: 89, 2022; DOI: 10.3892/ijmm.2022.5144

Following the publication of this paper, it was drawn to the Editor's attention by a concerned reader that certain of the flow cytometric data shown in Fig. 1D, DCFH-DA-stained cellular data in Fig. 2A, and western blotting data in Figs. 2G and 5B were strikingly similar to data that had either already appeared in previously published articles written by different authors at different research institutes (one of which has since been retracted), or were featured in an article that was submitted for publication to a different journal at around the same time. Owing to the fact that the contentious data in the above article had already been published prior to its submission to *International Journal of Molecular Medicine*, the Editor has decided that this paper should be retracted from the Journal. The authors were asked for an explanation to account for these concerns, but the Editorial Office did not receive a reply. The Editor apologizes to the readership for any inconvenience caused.

